# Kyste hydatique pulmonaire chez l'enfant traité par thoracoscopie: huit ans d'expérience

**DOI:** 10.11604/pamj.2013.15.96.1873

**Published:** 2013-07-12

**Authors:** Khalid Khattala, Aziz Elmadi, Mohamed Rami, Hanan Bouamama, Youssef Bouabdallah

**Affiliations:** 1 Service de chirurgie pédiatrique, CHU hassan II, Fès, Maroc

**Keywords:** Kyste hydatique, thoracoscopie, enfant, Maroc, hydatid cyst, thoracoscopy, child, Morocco

## Abstract

L’échinococcose kystique est une pathologie fréquente en zone d'endémie: pourtour méditerranéen, Afrique de l'est et l'Amérique du Sud. L'hydatidose reste à l'heure actuelle un problème majeur de santé publique. Notre travail consiste en une étude rétrospective de 27 malades opérés pour kyste hydatique pulmonaire (KHP) par thoracoscopie, au service de chirurgie pédiatrique du centre hospitalier universitaire HASSAN II à Fès, sur une période de huit ans allant de janvier 2004 au décembre 2011.

## Introduction

Le kyste hydatique pulmonaire chez l'enfant est fréquent en zone d'endémie, la thoracoscopie s'avère un moyen excellent avec beaucoup d'avantage pour traiter ce type de pathologie, nous allons rapporter une série de 27 malades avec 31 KHP traités par thoracoscopie tout en rapportant quelques différences techniques qui permettent de gagner un peu de temps pour la durée opératoire.

## Méthodes

Il s'agit d'une étude rétrospective à propos de 31 kystes hydatiques pulmonaires traités par voie thoracoscopique chez 27 malades, entre janvier 2004 et décembre 2011. Le recueil des données est fait d'après leurs dossiers médicaux et leurs suivis réguliers. Les paramètres suivants furent pris en compte: âge, symptomatologie, données radiologiques, techniques opératoires et évolution.

## Résultats

On note une prédominance féminine avec un sexe ratio égal à 0,7. L’âge de nos patients varie entre 5 et 15 ans, avec une moyenne d′âge de 8 ans, la tranche d′âge la plus touchée est comprise entre 10 et 15 ans, soit 54% des cas. Les patients d'origine rurale représentent 71% des cas, La notion de contage hydatique a été retrouvée chez 18 de nos patients (67%).

Les signes généraux sont dominés par la fièvre avec un pourcentage de 55% des cas, l'altération de l’état général (25,9%), la toux chronique est présente dans 62,9%; la douleur thoracique représente 55% et elle est en général basithoracique droite ou gauche; l'hémoptysie vient en troisième position avec 48% et elle se voit surtout en cas de KHP évolué; la vomique hydatique traduit la rupture du KHP dans les bronches et elle est retrouvée chez 4 malades soit 14,8% des cas; la dyspnée est retrouvée chez 18,5% des malades et elle est l'apanage des kystes volumineux ou compliqués. L'examen pleuropulmonaire trouve un syndrome d’épanchement liquidien chez 48,14% de nos patients; un syndrome d’épanchement aérique chez 3,7% de nos malades, un encombrement bronchique chez 18,5% des cas, et un seul cas d'hépatomégalie.

L'examen clinique était normal dans sa globalité chez 3 enfants soit 11,11%. L'hyperleucocytose est retrouvée chez 10 patients, soit 37% des cas. L'hyperéosinophilie est détectée chez 8 patients soit 29,62% des cas. Une anémie a été retrouvée chez 9 patients, soit dans 33,33% des cas. La vitesse de sédimentation réalisée chez 5 patients dans notre série. Elle était positive chez 3 malades et sa valeur était comprise entre 15 et 20 mm. La sérologie hydatique utilisant l'hémaglutination indirecte n'a été réalisée que chez 12 patients, soit 44,44% des cas. Elle s'est révélée positive avec dans 7 cas (58,33%).

Le nombre total de kystes recensés est 31, la taille moyenne des KHP est égale à 8 cm, avec des tailles extrêmes situées entre 4 et 12 cm; 90% des kystes opérés avaient une taille inférieure à 10 cm. On a retrouvé un KHP unique chez 23 malades soit 85,18%, un KHP multiple chez 4 malades soit 14,8%: la localisation unilatérale, a été retrouvée chez 2 patients touchant le poumon droit dans un cas et le poumon gauche dans un autre cas. Quant à l'atteinte bilatérale, elle a été retrouvée chez deux malades. Le KHP est situé à droite chez 18 malades soit 66,66% et à gauche chez 9 malades soit 33,33%. Le lobe inferieur droit est le plus touché chez 54,8% des cas, contre 16,12% pour le lobe inferieur gauche.

L’échographie thoracique est réalisée chez 2 patients, soit 7,4% des cas. En plus des lésions kystiques, l’échographie a permis de détecter un épanchement pleural droit enkysté avec une pachypleurite de la petite scissure droite, condensation parenchymateuse lobaire inférieure gauche. L’échographie abdominale est réalisée systématiquement chez tous nos patients à la recherche d'une localisation secondaire, elle a permis de montrer l'association de KHP + kyste hépatique représente 14,8%. Le scanner thoracique a été demandé chez 2 patients, soit 7,4% des cas permettant de confirmer le diagnostic.

Pour la période allant de janvier 2004 au décembre 2011, on a 142 malades hospitalisés pour cure de KHP dont 27 cas qui ont été traités par voie thoracoscopique et 115 cas traités par thoracotomie. Toutes les interventions ont été menées sous anesthésie générale et l'intubation orotrachéale était sélective chez 12 patients. Les malades sont installés en décubitus latéral. Les voies d'abord pour le traitement thoracoscopique varient selon le siège du KHP à traiter.

La protection du champ opératoire a été réalisée par des champs stériles imbibés de sérum salé hypertonique dans 20 cas (64%) et par injection direct dans la cavité pleurale dans 11 cas (36%). Le chirurgien est le plus souvent du côté du dos avec en face de lui son aide et le moniteur plus à droite, l'instrumentiste est à gauche du chirurgien près du chariot à instruments.

Nous avons utilisé un trocart de 10 mm pour optique introduit au niveau du 5ème espace intercostal sur la ligne médio-axillaire ou au niveau de la pointe de l'omoplate, qui va permettre dans un premier temps l'exploration de la cavité pleurale et la détermination du site d'emplacement des autres trocarts sous contrôle optique. Le deuxième temps opératoire a consisté en la mise en place de deux trocarts de 5 mm pour les instruments opérateurs. L'un des deux trocarts est situé à l'aplomb du KHP. Ces trois trocarts ont été toujours suffisants et ont toujours répondu à la règle de triangulation.

**Figure 1:**
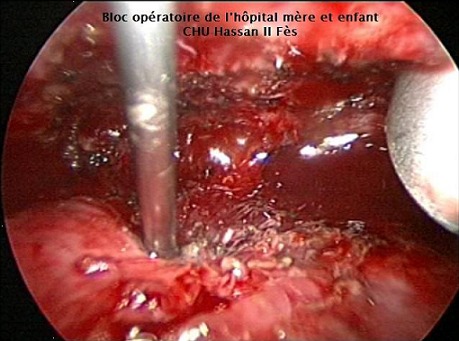
Ponction-injection et aspiration du liquide hydatique

**Figure 2:**
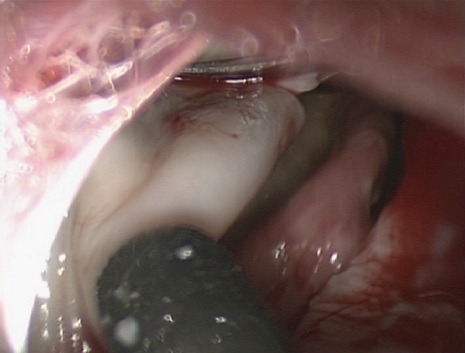
Résection du dôme saillant

**Figure 3:**
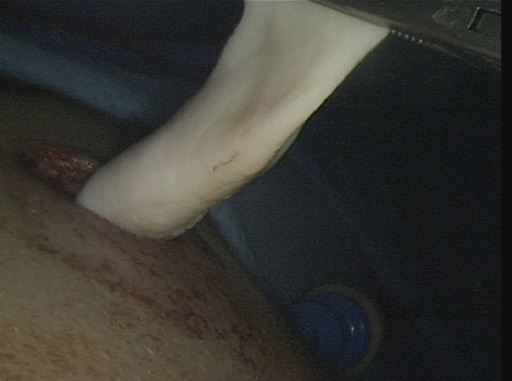
Extraction de la membrane proligère par aspiration à travers le trocart de 10 mm

Pour tous nos patients, la cure du KHP a débuté par l'exploration et la libération des adhérences, puis on a réalisé la ponction-aspiration du KHP avec injection de scolicide (sérum salé hypertonique) (Figure 1), la résection du dôme saillant à l'aide du ciseaux (Figure 2) et l'extraction de la membrane proligère à l'aide d'un petit sac en plastique pour 15 kystes (48,38%) ou par aspiration à travers l'orifice du trocart de 10 mm pour les autres 16 cas (51,6%) (Figure 3), puis on a réalisé la désinfection de la cavité résiduelle au sérum salé hypertonique, la recherche et le traitement des fistules bronchiques est faite après remise en circuit du poumon exclu sous faible pression pour repérer les fistules qui sont suturées par des points en X. Le capitonnage n’était fait que pour 10 kystes (32,25%). Le dernier temps opératoire est le drainage et la fermeture pariétale. La durée moyenne de l'intervention est de 90 minutes avec des extrêmes: 45-150 minutes. Le drainage thoracique était systématique pour tous les patients et la durée moyenne du drainage thoracique est de 24 heures pour les kystes de moins de 5cm et de 5 jours pour les kystes de plus de cette taille. Deux cas de KHP bilatéral ont été traités en deux temps opératoires sans incidents, avec un délai d'un mois entre les deux interventions. Les deux autres cas de KHP multiple unilatéral ont été traités en même temps opératoire.

La double localisation poumon-foie retrouvée chez 4 patients était traitée en 2 temps: traitement de la localisation pulmonaire en premier temps, suivie après un intervalle de 2-4 mois, d'un traitement du kyste hydatique du foie. Deux incidents peropératoires ont été enregistrés: Deux cas de conversion en thoracotomie classique vue la difficulté de libérer les adhérences importantes. Une seule situation de désaturation qui a obligé l’équipe chirurgicale à élargir la plaie en 05 centimètres.

Les suites opératoires étaient simples chez 85,18% des patients. Quatre malades ont présenté une infection de la cavité résiduelle dont 2 malades ont bien évolués et les deux autres ont été re-drainés et mis sous antibiothérapie pendant 15 jours. Sept malades ont présenté un emphysème sous cutané qui a rapidement régressé (25,9%). La moyenne de la durée totale d'hospitalisation est de 2 jours pour les kystes de moins de 5 cm et de 5 jours pour les kystes de plus de 5cm.

Le suivi post opératoire en consultation montre une disparition progressive de l'image résiduelle sur les radiographies thoraciques, aucune récidive n'a été notée sur un recul moyen de 36 mois allant d'un an à 5 ans.

## Discussion

La thoracoscopie était jusqu′aux années 1970 limitée à l′exploration de la cavité pleurale. Depuis, cette technique s′est développée parallèlement à la chirurgie cœlioscopique. En 1976, Rodgers a rapporté les premières thoracoscopies réalisées chez l′enfant. Il s′agissait alors d′interventions à visée diagnostique, où les gestes pratiqués étaient limités à des biopsies ganglionnaires ou pulmonaires [1]. La chirurgie thoracoscopique a acquis un vaste éventail d'indications opératoires chez l'enfant, y compris le nouveau-né [2].

Le traitement du KHP par une thoracoscopie s'avère une excellente alternative à la thoracotomie. Il n'y a pas de position standard pour l'introduction des trocarts. Le premier trocart est généralement introduit dans le 5ème espace intercostal sur la ligne axillaire moyenne, une incision est faite au bord supérieur de la cote, de taille très légèrement plus grande que le diamètre du trocart à introduire, le trajet est créé par une pince Kelly ou Kocher jusqu’à la plèvre [3, 4].

Ce premier trocart de 10 mm permet l'introduction du système optique relié à une caméra vidéo. Ce temps d'exploration va pouvoir commencer par déplacement de l'axe de l'optique dans diverses directions, toute la cavité thoracique sera visualisée. Il s'agit d'un temps essentiel, minutieux qui va permettre un repérage exact de la lésion, une visualisation précise des éventuelles adhérences. Deux autres trocarts de diamètre variable sont placés sous contrôle de la vue pour donner accès aux instruments; l'idéal est que les trois trocarts soient disposés en triangulation.

L'un des trocarts opérateurs est placé à l'aplomb du dôme du KHP de sorte que le trajet soit le plus court possible entre la paroi et le KHP; par ce trocart on introduit un trocart de ponction relié directement à l'aspiration. On procède donc à la ponction-évacuation du kyste; le calibre du trocart et la force de pression permettent en général de vider rapidement le contenu du kyste. On maintient dès lors le trocart dans le kyste sous aspiration, et on introduit une pince de préhension type endoduval pour suspendre le kyste, ainsi le trocart de ponction est retiré et des ciseaux coagulateurs sont introduits. On procède à l'agrandissement de l'ouverture du perikyste, et une canule d'aspiration est introduite par le trocart de ponction. On injecte le sérum salé hypertonique, du cetrimide ou de l’éthanol et après 10 à 15 min, on aspire le contenu et la membrane proligère est introduite dans le sac pour l'extraire [3, 4].

Le traitement du KHP, sous thoracoscopie ne doit pas déroger aux règles élémentaires du traitement réalisé par thoracotomie résumé par l'acrostiche PAIRE (ponction-aspiration-injection de scolicides-réaspiration). L'utilisation des scolicides dans la cavité pleurale libre remplace les compresses imbibées de ce même produit. Par la suite, on procède à la résection du dôme saillant. La recherche et le traitement de fistules bronchiques peut se faire sous contrôle vidéo. L'exérèse terminée, et le capitonnage n'est pas toujours nécessaire, le drain thoracique est mis en place au travers des orifices des canaux opérateurs principaux, le plus souvent, on draine par deux drains thoraciques. Finalement, fermeture des différents orifices restants.

La plupart des patients arrivent ex tubés en salle de surveillance post-interventionnelle; ils sont installés en position semi-assise, l'apport d'O2 est débuté, les drains thoraciques sont remis en aspiration. L'obtention d'une analgésie satisfaisante est un objectif important, permettant une kinésithérapie précoce. Les avantages de la thoracoscopie sont une durée d'intervention moindre, une analgésie réduite, une durée d'hospitalisation minimale [5, 6]. Au terme de notre étude on a remarqué que la durée d'hospitalisation est plus prolongée pour les kystes de plus de 5cm du faite que les fistules sont plus grosses et prennent du temps pour se colmater, pas de différence soit qu'on utilise des compresses imbibés de sérum hypertonique ou que l'on injecte directement dans la cavité pleurale, encore l'extraction de la membrane proligère peut se faire à l'aide d'un sac en plastique ou par aspiration directe à travers le trocart de 10mm.

## Conclusion

Le kyste hydatique du poumon est un véritable problème de santé publique. Le traitement chirurgical reste actuellement le seul traitement efficace, grâce à la vidéochirurgie thoracique on réalise à minima un geste thérapeutique complet et de ce fait et de part ces avantages, elle s'impose dans le traitement des KHP sains, petits et périphériques, et s'avère donc une excellente alternative à la thoracotomie conventionnelle, encore l'aspiration directe du kyste permets de gagner du temps opératoire sans risque de contamination de la paroi selon notre étude.
